# Shexiang Baoxin Pills Could Alleviate Isoproterenol-Induced Heart Failure Probably through its Inhibition of CaV1.2 Calcium Channel Currents

**DOI:** 10.1155/2022/5498023

**Published:** 2022-11-09

**Authors:** Jianwei Wu, Juan Yu, Jianyong Qi, Minzhou Zhang

**Affiliations:** ^1^Affiliated Hospital of Guangzhou University of Chinese Medicine, Guangdong Province Hospital of Chinese Medicine, Guangdong Province Academy of Chinese Medicine, Guangzhou 510006, China; ^2^Intensive Care Research Team of Traditional Chinese Medicine, Guangdong Province Hospital of Chinese Medicine, Guangdong Province Academy of Chinese Medicine, Guangzhou 510006, China; ^3^AMI Key Laboratory of Chinese Medicine in Guangzhou, Guangdong Province Hospital of Chinese Medicine, Guangdong Province Academy of Chinese Medicine, Guangzhou 510006, China

## Abstract

Heart failure (HF) affects millions of patients in the world. Shexiang Baoxin Pills (SXB) are extensively applied to treat coronary artery diseases and HF in Chinese hospitals. However, there are still no explanations for why SXB protects against HF. To assess the protective role, we created the HF model in rats by isoproterenol (ISO) subcutaneous injection, 85 milligrams per kilogram body weight for seven days. Four groups were implemented: CON (control), ISO (HF disease group), CAP (captopril, positive drug treatment), and SXB groups. Echocardiography was used to evaluate rats' HF *in vivo*. The human CaV1.2 (hCaV1.2) channel currents were detected in tsA-201 cells by patch clamp technique. Five different concentrations of SXB (5, 10, 30, 50, and 100 mg/L) were chosen in this study. The results showed that SXB increased cardiac systolic function and inhibited rats' cardiac hypertrophy and myocardial fibrosis induced by ISO. Subsequently, it was found that SXB was inhibited by the peak amplitudes of hCaV1.2 channel current (*P* < 0.01). The SXB half inhibitory dosage was 9.09 mg/L. The steady-state activation curve was 22.8 mV depolarization shifted; while the inactivation curve and the recovery from inactivation were not affected significantly. In conclusion, these results indicated that SXB inhibited ISO-induced HF in rats and inhibited the hCaV1.2 channel current. The present study paved the way for SXB to protect itself from HF.

## 1. Introduction

Heart failure (HF) causes hazardous illness globally. There were nearly 6.0 million Americans >20 years of age suffered HF from 2015 to 2018. [1] With the progress of society and the development of medical science and technology, more and more measurements were implemented to treat HF, for example, drug therapies including salkubatroxartan, hydrogen nitrate, *β*-blockers, angiotensin-converting enzyme inhibitors, and nondrug therapies, such as left ventricular assist device implantation, pacemaker implantation, and even heart transplantation. However, there was still extensive HF occur [2, 3]. During the 20 years from 1995 to 2015, although there was a significant decrease in ischemic heart disease, HF increased from 15.5 to 16.3 people per 10,000 general population in the US [1]. Therefore, novel alternative medical treatments could benefit patients with HF.

Shexiang baoxin pills (SXB), originated from the Suhexiang pill, were recorded in the world's first prescription pharmacopoeia—Taiping Huimin Heji prescription of the Song dynasty, nearly 1000 years ago. Since then, SXB has been extensively treating various diseases; including heart disease [4], brain ischemic disease [5], renal injury [6], and atherosclerosis [7]. Furthermore, SXB has been mostly used in cardiovascular diseases, including HF and angina pectoris [8–10]. Studies have elucidated that multiple signaling pathways participate in SXB's protection mechanisms, for example, mitogen-activated protein kinase (MAPK), phosphatidylinositol 3-kinase (PI3K) [11], cytochrome P450 [12], and 20-hydroxyeicosatetraenoic acid [13] participated in the process. SXB also inhibited lipid accumulation up-regulating, improving inflammation response and preserving energy metabolism, and so on. [14].

Although SXB is effective clinically, the mechanisms of SXB treating HF were unknown yet. To clarify the roles, the model of ISO-induced HF in rats was constructed and the changes in CaV1.2 currents were detected by the patch clamp method *in vitro*.

## 2. Materials and Methods

### 2.1. Animals and Reagents

All the animal procedures were implemented under the agreement of the Institutional Animal Care and Use Committee of Guangzhou University of Chinese Medicine, Guangdong Province Hospital of Chinese Medicine, and the guideline of the Care and Use of Laboratory Animals (Department of Health and Human Services, National Institutes of Health, Publication No. 86–23, revised 1996). Male SD rats were ordered from the Experimental Animal Center of Guangdong Province. Each rat weighed 200–300 gram. SXB was ordered from Shanghai Hutchison Pharmaceuticals Company (batch NO: 190601, Shanghai, China). SXB was prepared in accordance with the description provided in Chinese Pharmacopeia 2020 [15].

### 2.2. HF Model in Rats

We followed the methods of Qi et al. 2020 [16, 17]. Briefly, isoproterenol (ISO) was subcutaneously administered in rats for 7 days with a dosage of 85 mg/kg/day to construct an HF model. Four groups were assigned as followed: CON (control, *n* = 13), ISO (HF model, *n* = 13), CAP (captopril, *n* = 7), and SXB (*n* = 10). Saline was subcutaneously administered to the CON group; the ISO group was subcutaneously administered with ISO for 7 days. Captopril, which served as a positive control to treat HF, was subcutaneously administered at 20 mg/kg/day for 7 days after ISO stimulation in CAP rats. According to publications, our preliminary research, and conversion of the clinical humans' oral usage to rats [17], SXB powder was mixed with one-milliliter saline, at a dosage of 2.5 gram per kilogram body weight of rats, was gastric gavage daily after seven days of ISO stimulation.

### 2.3. Echocardiography

The linear array transducer (17.5 MHz) was inserted into an echocardiographic machine (Vevo 770, Visual Sonics, Toronto, Canada). Left ventricular (LV) structure and function were determined by detecting LV diastolic posterior wall thickness (LVPWd) and fraction shortening (FS). After anaesthetized by tribromoethanol intraperitoneal, rats were laid on a thermostatic plate. Firstly, at the papillary muscle level, the LV short-axis two-dimensional (2D) images were obtained, then 2D long-axis images and M-mode images were collected, and subsequently, LV end-diastolic or systolic inner dimension (LVIDd and LVIDs), LV end-diastolic anterior wall thickness (LVAWd), and LVPWd were detected. Other values could be obtained automatically by the echocardiography system, such as LV eject fraction (EF), end-diastolic/systolic volume (LVVd/s), and FS.

### 2.4. HW Detection and Immunohistochemistry

Rats were sacrificed, and the body weight (BW), heart weight (HW), and tibial length (TL) of rats were determined. Then, the whole hearts were arranged with paraffin section staining. Pathological sections were cut into 5 *μ*m slices. Myocyte hypertrophy was analyzed by staining with hematoxylin-eosin (HE). Cardiac fibrosis was evaluated by staining with Masson and Sirius red. The sizes of cardiomyocytes from each rat were detected with light microscopy at ×400 magnification. Myocardial fibrosis was quantitatively measured by a different color in Masson and Sirius red at ×40 magnifications. The data of hypertrophied myocytes and cardiac fibrosis were obtained with Image-Pro Plus software.

### 2.5. Cellular Experiments

The human Cav1.2 (hCaV1.2) cDNA (GeneBank accession number: NM_000710) was inserted into in pcDNA3.1-T2A-EGFP2 plasmid vector. The constructs were verified by direct DNA sequence analysis. Human tsA-201 cells (catalogue no. CBP61213), which originated from a human embryonic kidney cell line expressing a simian virus 40 T-antigen, were purchased from the European Collection of Authenticated Cell Cultures (ECACC, London, UK). The cellular cultured medium was made up of 10% fetal bovine serum (Gibco, Gibco, Waltham, MA), Dulbecco's modified Eagle's medium (Gibco, Waltham, MA), and 100 U/mL penicillin and 100 *μ*g/mL streptomycin. The lipofectamine 2000 (Invitrogen, Grand Island, NY, USA) was used to transfect hCav1.2 cDNA into the tsA-201 cell line as we described previously [18]. In short, the DNA/liposome mixture, which was made up of 2 *μ*g hCaV1.2 plasmid, 8 *μ*l lipofectamine-2000 (Invitrogen, Grand Island, NY), and 0.8 mL Opti-MEM (Invitrogen, Grand Island, NY), transfected into the 50–70% confluence tsA-201 cells in 35 mm dishes for 12–24 hours, subsequently replaced the mixed medium by normal cell culture medium. It could be maintained for 1–3 days for commencing electrophysiological recordings.

### 2.6. Cellular Electrophysiological Recording

PC-100 horizontal microelectrode puller (NARISHIGE Co., Japan) was used to pull the borosilicate glass electrode into the patch pipette. When filled with intracellular fluid, the tip resistance of the pipette varied between 3 and 5 MΩ. An EPC-10 amplifier, combined with Pulse software (HEKA, Lambrecht, Germany), was used to record the membrane currents in a voltage-clamp mode. Firstly, to offset the liquid junction potentials between bath and pipette solutions. Next, to form a gigaohm seal, gentle suction, rupture the cell membrane, and stand quietly for 3 minutes to form a stable steady-state whole-cell configuration. Whole-cell capacitive currents were compensated, leak subtracted, and series resistance was compensated to 60–80%. 10 kHz and 2.9 kHz were used to sample and filter the current signal respectively (8-pole Bessel filter, 3 dB). A laptop was used to record and store the current signal. Room temperature was kept at 22–24°C for all the experiments implemented.

### 2.7. Drugs and Solutions

After a gentle vortex, SXB was dissolved and filtered in sterile distilled water, with the concentration of the stock solution at 10 mg/mL. Subsequently, the targeted bath solution was obtained by diluting the stock solution with an extracellular solution. As for detecting the hCav1.2 channel currents, the pipette solution contained five reagents as followed (in mM) 42 HEPES, 4 Mg-ATP, 120 NMDG-Cl, 5 EGTA, and 1 MgCl_2_ (the pH value was adjusted to 7.3 by methane sulfonic acid). The bath solution contained three reagents as followed (in mM) 105 Tris, 1 MgCl_2_, and 40 BaCl_2_ (the pH value was adjusted to 7.3 by methane sulfonic acid) [19]. The bath solution was changed and the tsA-201 cells were continually perfused with a constant flow rate of one to two milliliters per minute [20]. Signal currents were recorded after the whole-cell configuration formed at least three minutes so as to make the pipette solution undergo complete dialysis.

### 2.8. Analyses of the hCaV1.2 Channel Currents and Statics

Origin 8.0 software (Origin Lab Corp., Northampton, MA, USA) and Patch Master (HEKA Electronics, Lambrecht/Pfalz, Germany) were used to collect and analyze the hCaV1.2 channel current data. The Boltzmann equation was used to fit the activation curves as follows:

(1) $$\begin{array}{c} {Fraction\;of\;maximal\;current=\left[1+\exp\left[\frac{-\left(V_{t}-V_{a}\right)}{K_{a}}\right]\right]}^{-1}\text{,} \end{array}$$

where, *V*_*t*_ is the test potential, *V*_*a*_ is the half-activation potential of the hCaV1.2 channel conductance, and *k*_*a*_ is the slope factor in the hCaV1.2 activation stage.

The curves of the steady-state inactivation with and without SXB perfusion were fitted with the equation as follows:

(2) $$\begin{array}{c} \frac{\left({I-I}_{c}\right)}{\left(I_{\max}-I_{C}\right)}={\left[1+\exp\frac{\left(V_{t}-V_{i}\right)}{K_{i}}\right]}^{-1}\text{,} \end{array}$$

where, *I*_max_ is the maximum current from the absence of inactivation, and *I*_*c*_ is a noninactivating current. *V*_*i*_, *V*_*t*_, and *K*_*i*_ are the half-inactivated potential, the half-inactivated slope factor, and the test potential, respectively.

For statistical analysis, data were collected as means ± standard error of the means (SEM). An unpaired *t*-test was used to compare two groups, while multiple groups (three or more than three groups) were compared by one-way analysis of variance plus the Bonferroni test. Significant differences were accepted when *P* values less than 0.05.

## 3. Results

### 3.1. SXB Increased Cardiac Function in HF Rats

**Figure 1:**
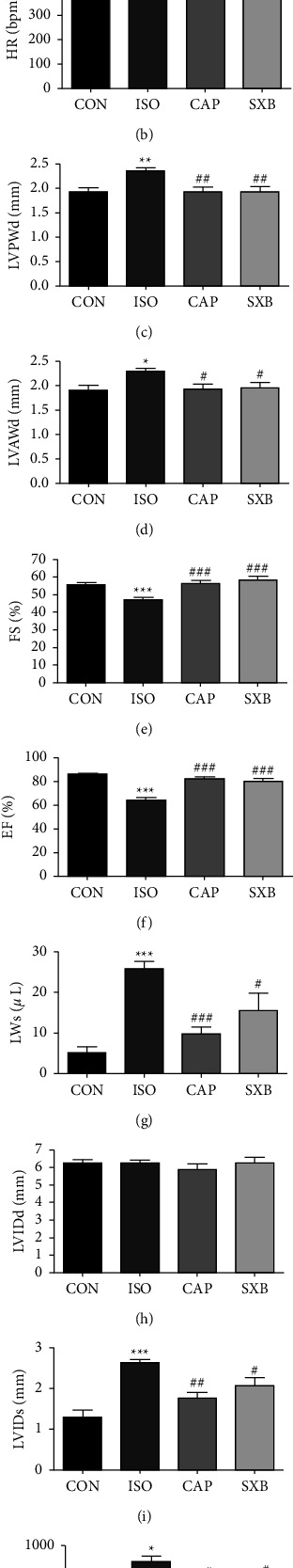
Echo data from the 4 groups. (a) Typical echo screenshots of three consecutive cycles. (b) HR (c) LVPWd (d) LVAWd (e) FS (f) EF (g) LVVs (h) LVIDd (i) LVIDs (j) LVMc were measured among the CON (*n* = 13), ISO (*n* = 13), CAP (*n* = 7), and SXB groups (*n* = 9). ^*∗*^*P* < 0.05, ^*∗∗*^*P* < 0.01, ^*∗∗∗*^*P* < 0.001, vs. the CON group. ^#^*P* < 0.05, ^##^*P* < 0.01, ^###^*P* < 0.001, vs. the ISO group.

A typical figure of the rat HF model induced by ISO is exhibited in Figure 1(a) with a 1-mode LV echo imaging. As shown in Table 1 and Figure 1(b), heart rates (HRs) were not changed among the CON (*n* = 13), ISO (*n* = 13), CAP (*n* = 7), and SXB groups (*n* = 9, *P* > 0.05). Both LVPWd (Figure 1(c)) and LVAWd (Figure 1(d)) were increased, while FS (Figure 1(e)) and EF (Figure 1(f)) were both reduced in ISO group (LVPWd, ISO, 2.30 ± 0.06 millimeter(mm); CON, 1.92 ± 0.09 mm, *P* < 0.01; FS, ISO, 47.26 ± 1.21%; CON, 55.89 ± 1.16%, *P* < 0.001, respectively). LVVs of the ISO group were increased (LVVs, ISO vs. CON, *P* < 0.001, Figure 1(g)). So, decompensate cardiac hypertrophy existed in the ISO group. To ensure the roles of SXB on HF, LVPWd was compared with the ISO group, and it showed that LVPWd were reduced in both CAP and SXB groups (Table 1, CAP, 1.93 ± 0.09 mm vs. SXB, 1.93 ± 0.10 mm, *P* < 0.01), yet cardiac systolic function was augmented in CAP and SXB groups (FS, CAP, 56.70 ± 1.33%, SXB: 58.57 ± 1.89%, *P* <  0.01, vs. ISO group). Together, the study showed that captopril and SXB improved cardiac systolic function and reversed eccentric cardiac hypertrophy in HF rats.

### 3.2. SXB Inhibited HF After ISO Stimulation in Vivo

**Figure 2:**
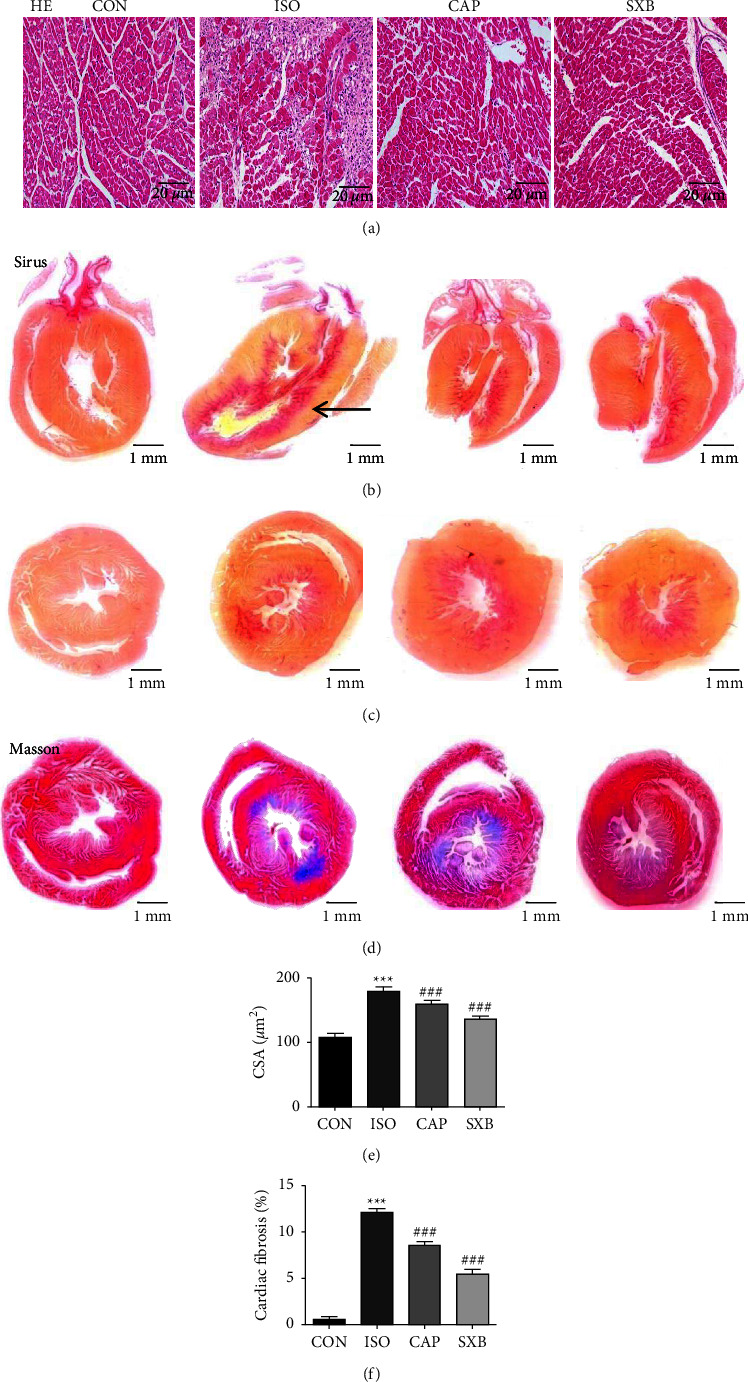
Pathological sections among the CON, ISO, CAP, and SXB groups. (a) HE-stained, (b) long-axis Sirius red-stained, (c) short-axis Sirius red-stained, and (d) Masson-stained heart sections among the CON (*n* = 13), ISO (*n* = 13), CAP (*n* = 7), and SXB groups (*n* = 9). (e) Mean CSA of cardiomyocytes and (f) the fraction of fibrotic area. ^*∗∗∗*^*P* < 0.001, vs. the CON group. ^###^*P* < 0.001, vs. the ISO group.

To further ensure the role of SXB effects on HF, microscopic anatomic analyses were performed on HE, Sirius red, and Masson-stained thin sections of hearts. Moreover, the heart, lung, and liver after the rats were sacrificed, were weighed and statistically compared to the CON, ISO, CAP, and SXB groups.  Figure 2(a) shows that the cross-section area (CSA) of the ISO group was more augmented than CON rats (Figure 2(e), ISO, 198.60 ± 1.46 *μ*m^2^ vs. CON, 109.30 ± 0.78 *μ*m^2^, *P* < 0.001), while it was reduced in CAP and SXB rats (CAP, 161.50 ± 0.76 *μ*m^2^ vs. SXB, 137.80 ± 0.57 *μ*m^2^, respectively). Figures 2(b) and 2(c) are the long-axis and short-axis of Sirius-stained hearts sections of the 4 groups respectively (cardiac fibrosis was indicated by the arrow).  Figure 2(d) is the typical Masson-stained heart section of the CON, ISO, CAP, and SXB groups. As shown in Figure 2(f), the area of myocardial fibrosis in the ISO group are more increased than in the CON rats' (12.12 ± 0.42% vs. 0.58 ± 0.29%, *P* < 0.001), while it is decreased in CAP and SXB rats (8.54 ± 0.42% vs. 5.41 ± 0.54%, respectively). Together, the study showed that SXB reversed pathological eccentric hypertrophy and myocardial fibrosis in rats with heart failure.

**Figure 3:**
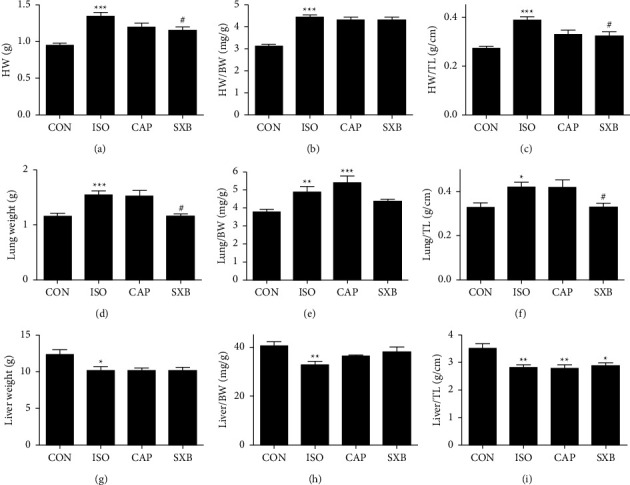
Anatomic data among the CON, ISO, CAP, and SXB groups. (a) HW, (b) HW/BW, (c) HW/TL, (d) lung weight, (e) lung/BW, (f) lung/TL, (g) liver weight, (h) liver/BW, and (i) liver/TL were compared among the CON (*n* = 13), ISO (*n* = 13), CAP (*n* = 7), and SXB groups (*n* = 9). ^*∗*^*P* < 0.05, ^*∗∗*^*P* < 0.01, ^*∗∗∗*^*P* < 0.001, vs. the CON group. ^#^*P* < 0.05, ^##^*P* < 0.01, ^###^*P* < 0.001, vs. the ISO group.

As shown in Figure 3(a) and Table 2, HW is significantly enhanced in the ISO rats (1.35 ± 0.05 g) than in the CON rats (0.95 ± 0.03 g, *P* < 0.001). The ratio of heart weight to body weight (HW/BW, Figure 3(b)) and the ratio of HW to TL (HW/TL, Figure 3(c)) were both augmented in ISO rats (HW/BW, ISO, 4.43 ± 0.12 milligram per Gram (mg/g) vs. CON, 3.11 ± 0.09 mg/g, *P* < 0.001; HW/TL, ISO, 0.39 ± 0.02 gram per centimeter (g/cm) vs. CON, 0.27 ± 0.01 g/cm, *P* < 0.001). Moreover, the lung weight (Figure 3(d)), the ratio of lung weight to body weight (Lung/BW, Figure 3(e)), and the ratio of lung weight to TL (Lung/TL, Figure 3(f)) were enhanced in ISO rats (lung weight: CON, 1.15 ± 0.06 g vs. ISO, 1.54 ± 0.08 g, *P* < 0.001; Lung/TL: CON, 0.33 ± 0.02 g/cm vs. ISO, 0.422 ± 0.02 g/cm, *P* < 0.05, respectively). However, the liver weight (Figure 3(g)), the ratio of liver weight to body weight (liver/BW, Figure 3(h)), and the ratio of liver weight to TL (liver/TL, Figure 3(i)) were decreased in the ISO group (*P* < 0.05, vs. CON), which revealed that ISO stimulation caused rat's heart failure with the pathological process of pulmonary hyperemia, yet without systemic hyperemia. Next, HW, the ratio of HW/BW, and HW/TL were compared among the ISO, CAP, and SXB groups. In Figure 3, HW and HW/TL are reduced in SXB rats (HW, 1.20 ± 0.06 g, HW/TL, 1.15 ± 0.05 g/cm) compared with ISO rats (HW, 1.35 ± 0.05 g, HW/TL, 0.39 ± 0.02, *P* < 0.05). Also, the lung weight and lung//TL were significantly reduced in SXB rats (lung weight, 1.16 ± 0.04 g, lung/TL, 0.33 ± 0.02 g/cm) compared with ISO rats (lung weight, 1.54 ± 0.08 g, lung/TL, 0.42 ± 0.02 g/cm, *P* < 0.05). Together, this research showed that SXB alleviated left ventricular failure after ISO stimulation.

### 3.3. SXB Inhibited the CaV1.2 Calcium Channel Current in a Dose-Denpendent Manner

**Figure 4:**
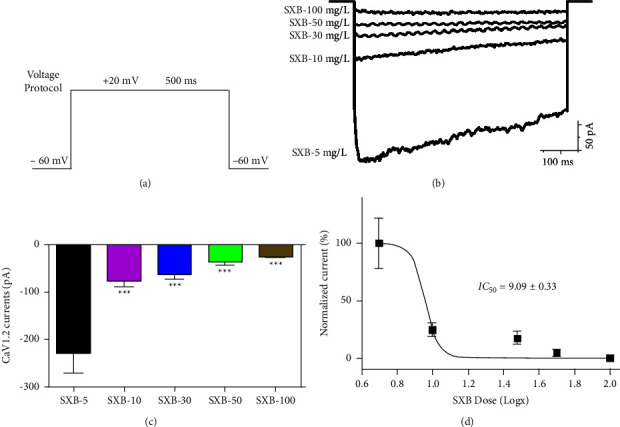
SXB dose-dependently inhibits the hCaV1.2 currents. (a) Voltage protocol was listed in detail in results 3.3. (b) Typical hCaV1.2 currents were represented in the 5 SXB groups (5.10, 30, 50, and 100 mg/L). Concentration-response bar graph (c) and curve (d) were displayed in 5 SXB groups and IC50 was listed. ^*∗∗∗*^*P* < 0.01, vs. the SXB −5 mg/L group.

To explore the unveiled nature of SXB's prevention from HF, the L-type calcium channel, the vital molecule in the calcium-induced calcium release (CICR) process, which participated in HF, were detected [21]. By whole cell patch clamp recording, we detected the hCaV1.2 channel currents, which is the main *α*-subunit of the L-type calcium channel. The voltage protocol is listed in Figure 4(a), which holding potential kept at −60 mV, depolarized to +20 mV for 500-ms.  Figure 4(b) shows the representative plots of the hCaV1.2 current curves with the five different SXB concentrations (5 mg/L, 10 mg/L, 30 mg/L, 50 mg/L, and 100 mg/L). As shown in Figure 4(c), the four SXB groups reduced the hCaV1.2 peak amplitude significantly, compared with the 5 mg/L SXB group (*P* < 0.001).  Figure 4(d) shows the dose-response curve of SXB affected by the hCaV1.2 current, with the half inhibitory concentration (IC_50_) as 9.09 ± 0.33 mg/L.

### 3.4. SXB Effects on the Gating Kinetics of the hCaV1.2 Channel Current

**Figure 5:**
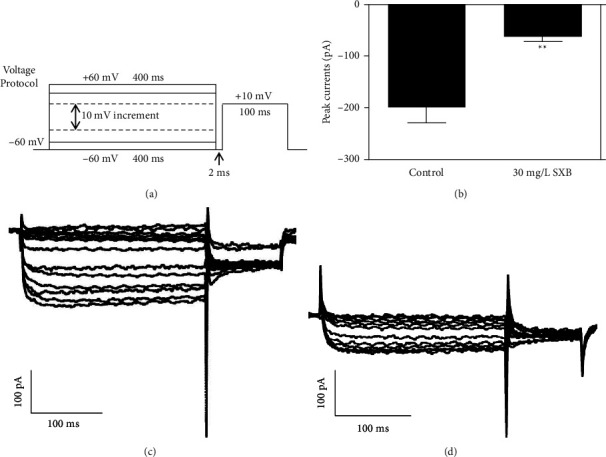
The hCaV1.2 currents were detected before and after 30 mg/L SXB perfusion in tsA-201 cells. (a) Voltage protocol was listed in detail in results 3.4. (b) Peak currents were compared between 30 mg/L SXB (*n* = 14) and control (*n* = 24). ^*∗∗*^*P* < 0.01, vs. the CON group. hCaV1.2 channel currents were detected before (c) and after (d) 30 mg/L SXB perfusion.

To further reveal the electrophysiological properties of SXB on the hCaV1.2 channel current, we analyzed the activation and inactivation curves.  Figure 5(a) shows the voltage protocol. The holding potential was −60 mV, the depolarizing pulses lasted for 400-ms, enhanced from −60 mV to +60 mV, with 10 mV increments per pulse, followed with a 2 ms interval, kept at −60 mV, then the test pulse was set to +10 mV for 100 ms, the total protocol was evoked every 15 seconds. [22, 23] Figures 5(c) and 5(d) are the typical hCaV1.2 currents curves before and after the SXB perfusion.  Figure 5(b) shows that the peak current of the hCaV1.2 channel was significantly reduced in the 30 mg/L SXB group, compared with the control (Ipeak, Control, −199.82 ± 30.39 pA, SXJ, −62.36 ± 10.67 pA, *P* < 0.01) in tsA-201 cells.

**Figure 6:**
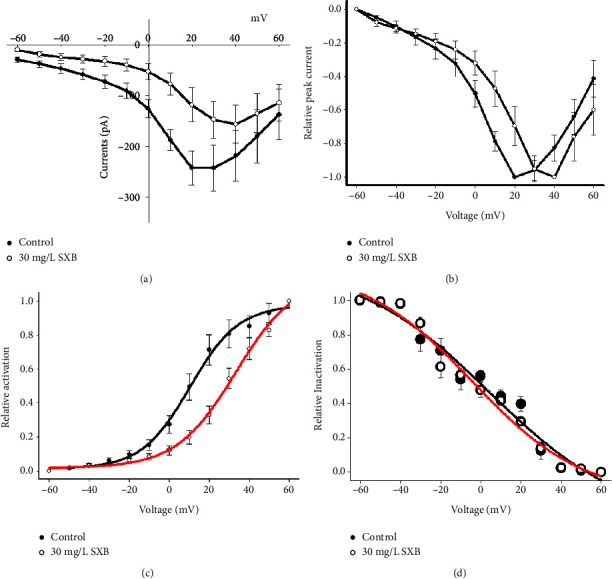
The activation and inactivation curves of hCaV1.2 currents with and without 30 mg/L SXB perfusion. (a) The steady-state activation curve, (b) relative peak current curve, (c) relative activation, and (d) inactivation curves of control (*n* = 11) and 30 mg/L SXB (*n* = 7) were obtained from hCaV1.2 current amplitudes.

The activation gating kinetics were compared and shown in Figure 6(a), a clear inhibition of hCaV1.2 currents was observed with 30 mg/L SXB. The characteristics of the I-V relationship showed that 30 mg/L SXB reduced the peak values of hCaV1.2 currents, which were reduced to 31.21% of the control, SXB changed the voltage of peak activation of the hCaV1.2 currents (the normalized I-V curves, Figure 6(b)). The activation curve of the current was shifted by approximately 23 mV toward the depolarized potential during cell exposure to SXB; however, no apparent change in the slope factor (i.e., Ka) was demonstrated in its presence (Figure 6(c)), which might be due to no altering the hCaV1.2 activation gating kinetics.

The inactivation curves were obtained with the above protocol (Figure 6(a)). The curves were obtained before and after 30 mg/L SXB perfusion (Figure 6(d)). The smooth curve was fitted to the Boltzmann equation (Methods. 2.8). No significant changes were shown between CON and SXB treating groups (*P* > 0.05). Together, the study showed that SXB did not influence the inactivated channels significantly in the present study.

### 3.5. SXB Effects on the Recovery from Inactivation (RFI) of the hCav1.2 Channel

**Figure 7:**
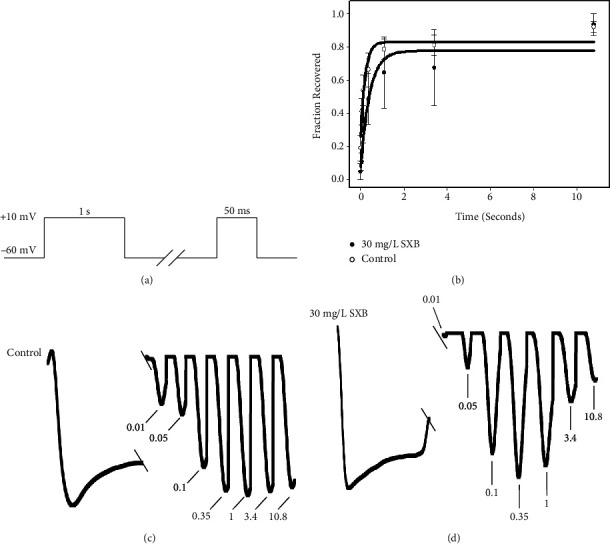
The RFI changes of the hCaV1.2 channel before and after SXB perfusion. (a) A voltage protocol was detailed mentioned in the results 3.5. (b) RFI curves were plotted from the (c) control (*n* = 4) and (d) 30 mg/L SXB (*n* = 6).

The RFI of hCav1.2 was detected with a double-pulse RFI protocol before and after SXB perfusion (Figure 7(a)). A 1s-pulse rose to +10 mV and then, decreased to a holding potential (−60 mV), followed by time intervals, changing from 50 ms to 10 s. After that, the potential again rose to +10 mV for 50 ms. Figures 7(c) and 7(d) were the typical RFI curves with or without SXB perfusion, which did not show significant differences between both the groups (Figure 7(b)). Thus, SXB did not regulate the extent of calcium channel recovery, regardless of the duration of pulse stimulation.

## 4. Discussion

The present study first explored the cardio-protective effects of SXB against ISO-induced HF in rats. The results demonstrate that SXB markedly reversed eccentric cardiac hypertrophy, decreased cardiac fibrosis, and increased cardiac systolic function in ISO-induced HF rats. Furthermore, SXB might regulate the hCaV1.2 currents.

There are various methods to construct animal HF models, such as transverse aortic constriction (TAC), spontaneously hypertensive rats, and ISO stimulation [24]. Constructing an HF model by using spontaneously hypertensive rats is time-consuming. Although it is commonly used to induce eccentric cardiac hypertrophy by the TAC method due to a high-pressure overload [25], it activates multiple signaling pathways, complicating mechanistic studies In contrast, ISO stimulation-induced HF is more maneuverable and can provide a more distinct mechanism. In our study, ISO-induced pathological myocardial hypertrophy is feasible. Although this method is not in common use, our studies revealed that ISO stimulation can be a convenient and stable method to construct a rat's model of decompensate cardiac hypertrophy.

We demonstrated that administration of ISO for 7 days induced heart failure, which was evaluated by an increase in LVVs in echocardiography(Figure 1(g)) as well as lung edema in anatomic data (Figures 2(d)–2(f)), SXB could reverse pathological eccentric cardiac hypertrophy and alleviate heart failure.

The HF mechanisms are complex and far more to elucidate clearly due to thousands of molecular pathways, such as a *β*-adrenergic receptor, L-type calcium channel, protein kinases (PK) A, PKC, Ca^2+^/calmodulin-dependent kinase II, calcineurin, and phosphodiesterase [26]. *β*-adrenergic stimulation can lead to HF, while the *β*-adrenergic antagonists can significantly alleviate heart failure [27, 28]. The mechanism underlying the altered CaV1.2 kinetics in HF is multilevel and multifactorial. The CaV1.2 channel is influenced by lots of molecules, activation of *β*-adrenergic receptors increases CaV1.2 current [29], increased sarcoplasmic reticulum [Ca^2+^], and augmented cardiac contractility. There are a lot of diseases occurring due to dysfunction of the CaV1.2 current, such as HF, cardiac hypertrophy, atrial fibrillation, and ischemic heart disease [30, 31]. It is vital for the L-type calcium channel current to activate the CICR in response to membrane depolarization in the heart. At least three subunits are essential to form the cardiac CaV1.2 channel: the pore-forming subunit (*α*1c) and two accessory subunits (*β*2a and *α*2/*δ*). Ca^2+^ influx was increased in transgenic mice overexpression of CaV1.2 *α*1C, which resulted in blunting of *β*-adrenergic modulation, cardiac hypertrophy, and HF [32–34]. Overexpressing the *β*2a subunit of CaV1.2 current, also augmented murine cardiac CaV1.2 activity, pathological hypertrophy, and increased death [35]. Therefore, over CaV1.2 current is harmful and can promote heart failure. Inhibiting the over CaV1.2 current is a strategy to treat HF. In our study, it was revealed that SXB could block the CaV1.2 channel current so as to alleviate HF.

Since SXB is made up of multiple compositions of herbs, such as artificial bezoar, artificial musk, borneol, cinnamon, ginseng extract, styrax, and venenum bufonis, it is difficult to elucidate the precise mechanism of each component of SXB. Furthermore, whether SXB influences other potassium or sodium channels, is still a mystery. Future works might be focused on the effects of SXB on other ion channels if it is possible.

In general, our study showed, for the first time, that SXB preferentially binds to the pore gate domain of hCaV1.2 so as to block the hCaV1.2 channels current, reversing the eccentric pathological hypertrophy, reducing myocardial fibrosis, and augmenting the cardiac function so as to treat HF. Therefore, the present mechanistic study paved the way for SXB protection from HF in clinics.

## Figures and Tables

**Table 1:** Echo values among the 4 groups (CON, ISO, CAP, and SXB).

	CON	ISO	CAP	SXB
HR (bpm)	490.90 ± 13.90	444.30 ± 10.80	451.10 ± 13.60	447.10 ± 9.74
LVAWd (mm)	1.90 ± 0.10	2.29 ± 0.06^*∗*^	1.93 ± 0.10^#^	1.95 ± 0.11^#^
LVAWs (mm)	3.53 ± 0.26	3.45 ± 0.11	3.34 ± 0.26	3.69 ± 0.17
LVIDd (mm)	6.22 ± 0.22	6.27 ± 0.15	5.87 ± 0.32	6.26 ± 0.33
LVIDs (mm)	1.29 ± 0.18	2.63 ± 0.08^*∗∗∗*^	1.77 ± 0.13^##^	2.07 ± 0.20^#^
LVPWd (mm)	1.92 ± 0.09	2.30 ± 0.06^*∗∗*^	1.93 ± 0.09^##^	1.93 ± 0.10^##^
LVPWs (mm)	3.56 ± 0.24	3.38 ± 0.07	3.41 ± 0.26	3.51 ± 0.15
EF (%)	86.21 ± 1.27	64.51 ± 1.90^*∗∗∗*^	82.21 ± 1.57^###^	80.57 ± 1.82^###^
FS (%)	55.89 ± 1.16	47.26 ± 1.21^*∗∗∗*^	56.70 ± 1.33^###^	58.57 ± 1.89^###^
LVM (mg)	81.98 ± 6.70	108.80 ± 5.56^*∗*^	81.49 ± 9.23^#^	84.77 ± 6.78^#^
LVMc (mg)	65.59 ± 5.36	87.01 ± 4.45^*∗*^	64.53 ± 7.23^#^	66.81 ± 5.81^#^
LVVd (mm^3^)	197.80 ± 15.40	200.70 ± 11.30	175.10 ± 21.40	203.20 ± 24.90
LVVs (mm^3^)	5.19 ± 1.39	25.75 ± 1.84^*∗∗∗*^	9.71 ± 1.78^#^	15.49 ± 4.33^#^

*Notes.* ^*∗*^ *P* < 0.05, ^*∗∗*^*P* < 0.01, ^*∗∗∗*^*P* < 0.001 vs. CON; ^#^*P* < 0.05, ^##^*P* < 0.01, ^###^*P* < 0.001 vs. ISO.

**Table 2:** Anatomic data among CON, ISO, CAP, and SXB groups.

	CON	ISO	CAP	SXB
Number (*n*)	13	13	7	9
BW (g)	305.30 ± 7.52	311.20 ± 13.50	278.10 ± 12.30	267.30 ± 9.43^#^
HW (g)	0.95 ± 0.03	1.35 ± 0.05^*∗∗∗*^	1.20 ± 0.06	1.15 ± 0.05^#^
Lung (g)	1.15 ± 0.06	1.54 ± 0.08^*∗∗∗*^	1.52 ± 0.11	1.16 ± 0.04^#^
Liver (g)	12.33 ± 0.66	10.12 ± 0.57^*∗*^	10.09 ± 0.42	10.11 ± 0.48
TL (cm)	3.52 ± 0.08	3.57 ± 0.09	3.64 ± 0.05	3.52 ± 0.10
HW/BW (mg/g)	3.11 ± 0.09	4.43 ± 0.12^*∗∗∗*^	4.30 ± 0.14	4.31 ± 0.12
Lung/BW (mg/g)	3.77 ± 0.14	4.88 ± 0.31^*∗∗*^	5.40 ± 0.38^*∗∗∗*^	4.35 ± 0.15
Liver/BW (mg/g)	40.39 ± 1.79	32.73 ± 1.62	36.33 ± 0.58	38.09 ± 2.08
HW/TL (g/cm)	0.27 ± 0.01	0.39 ± 0.02^*∗∗∗*^	0.33 ± 0.02	0.32 ± 0.02^#^
Lung/TL (g/cm)	0.33 ± 0.02	0.42 ± 0.02^*∗*^	0.42 ± 0.03	0.33 ± 0.02^#^
Liver/TL (g/cm)	3.51 ± 0.17	2.80 ± 0.11^*∗∗*^	2.78 ± 0.14^*∗∗*^	2.87 ± 0.11^*∗*^

*Notes.* ^*∗*^ *P* < 0.05, ^*∗∗*^*P* < 0.01, ^*∗∗∗*^*P* < 0.001 vs. CON; ^#^*P* < 0.05, ^##^*P* < 0.01, ^###^*P* < 0.001 vs. ISO.

## Data Availability

The data used to support the findings of this study are available from the corresponding author upon request.

## Abbreviations

CON: Control
CAP: Captopril
ISO: Isoproterenol
HR: Heart rates
HW: Heart weight
HF: Heart failure
BW: Body weight
TL: Tibial length
HE: Hematoxylin-eosin
FBS: Fetal bovine serum
FS: Fraction shortening
SXB: Shexiang baoxin pills
CSA: Cross-section area
LV: Left ventricle
EF: LV eject fraction
LVM_c_: LV corrected mass
LVV_d/s_: LV end-diastolic/systolic volume
LVPW_d_: LV diastolic posterior thickness
LVAW_d_: LV diastolic anterior thickness
LVID_d_: LV diastolic inner dimension
*V*_t_: Test voltage potential
*V*_a/i_: Half activated/inactivated voltage
*K*_a_: Slope factor for activated curve
*I*_max_: Maximum current of inactivation
*I*_c_: A noninactivating current
SEM: Standard error of the means
hCaV1.2: Human CaV1.2 calcium channel
MAPK: Mitogen activated protein kinase
PI3K: Phosphatidyl inositol 3-kinase
CICR: Calcium induced calcium release
RFI: Recovery from inactivation
TAC: Transverse aortic constriction.
